# Chlorotoxin-Fc Fusion Inhibits Release of MMP-2 from Pancreatic Cancer Cells

**DOI:** 10.1155/2014/152659

**Published:** 2014-01-06

**Authors:** Samah El-Ghlban, Tomonari Kasai, Tsukasa Shigehiro, Hong Xia Yin, Sreeja Sekhar, Mikiko Ida, Anna Sanchez, Akifumi Mizutani, Takayuki Kudoh, Hiroshi Murakami, Masaharu Seno

**Affiliations:** Division of Chemistry and Biotechnology, Graduate School of Natural Science and Technology, Okayama University, Okayama 7008530, Japan

## Abstract

Chlorotoxin (CTX) is a 36-amino acid peptide derived from *Leiurus quinquestriatus* (scorpion) venom, which inhibits low-conductance chloride channels in colonic epithelial cells. It has been reported that CTX also binds to matrix metalloproteinase-2 (MMP-2), membrane type-1 MMP, and tissue inhibitor of metalloproteinase-2, as well as CLC-3 chloride ion channels and other proteins. Pancreatic cancer cells require the activation of MMP-2 during invasion and migration. In this study, the fusion protein was generated by joining the CTX peptide to the amino terminus of the human IgG-Fc domain without a hinge domain, the monomeric form of chlorotoxin (M-CTX-Fc). The resulting fusion protein was then used to target pancreatic cancer cells (PANC-1) *in vitro*. M-CTX-Fc decreased MMP-2 release into the media of PANC-1 cells in a dose-dependent manner. M-CTX-Fc internalization into PANC-1 cells was observed. When the cells were treated with chlorpromazine (CPZ), the internalization of the fusion protein was reduced, implicating a clathrin-dependent internalization mechanism of M-CTX-Fc in PANC-1 cells. Furthermore, M-CTX-Fc clearly exhibited the inhibition of the migration depending on the concentration, but human IgG, as negative control of Fc, was not affected. The M-CTX-Fc may be an effective instrument for targeting pancreatic cancer.

## 1. Introduction

Pancreatic cancer is the fourth most common cause of cancer-related mortality worldwide [1] and is characterized by local invasion, early metastasis, and a strong desmoplastic reaction [2]. Blockage of the metastasis process remains a significant clinical challenge requiring innovative therapeutic approaches. For this purpose, molecules that inhibit matrix metalloproteinase (MMP) activity or induce the expression of their natural inhibitors, the tissue inhibitors of metalloproteinases (TIMPs), are potentially interesting [3]. Many MMP inhibitors have been developed for human clinical trials, but effective candidates have not yet been identified [4, 5]. *In vitro* studies have demonstrated that the proteolytic degradation of extracellular matrix (ECM) components is a major step in tumor invasion [6]. Among the enzymes involved in ECM degradation, the MMP family that contains at least 25 members of metzincin endopeptidases is the most studied. These enzymes are able to degrade ECM components [7–9]. MMPs are further divided into two subgroups based on whether the enzyme is either secreted or expressed on the cell surface in a membrane-tethered form soluble MMPs and membrane type MMPs (MT-MMPs) [10]. Soluble MMPs are secreted from cells into the extracellular milieu and can diffuse to distal sites. Therefore, it is believed that this type of MMP is useful for the degradation of ECM in a wider area [11, 12]. Because collagen IV is one of the major components of the basement membrane, MMP-2, a 72 kDa type IV collagenase, is believed to be of special significance during tumor invasion [2, 13].

MMP-2 is secreted as a proenzyme (proMMP-2) and located on the cell surface of tumor cells and requires activation to exert its catalytic activation [2, 14]. MT1-MMP is expressed as a 63 kDa protein on the surface of tumor cells and acts as a cell-surface receptor and activator of proMMP-2 [15]. MT1-MMP on the cell surface is replenished by clathrin-dependent internalization, and its concentration is stabilized by TIMP-2 [16, 17].

Chlorotoxin (CTX) is a 36-amino acid peptide which contains four disulfide bridges and is derived from *Leiurus quinquestriatus* (scorpion) venom. Early studies demonstrated that CTX can inhibit a potentially glioma-specific chloride ion channel [18]. CTX is believed to bind a lipid raft-anchored complex that contains MMP-2 [19], membrane type-1 MMP, tissue inhibitor of metallopreotease-2 [20], and other proteins [21]. In addition to glioma cells, CTX has been shown to specifically bind to other tumors of neuroectodermal origin [22]. It was recently found that CTX not only binds a wide range of tumor cell types but is also internalized by proliferating human vascular endothelial cells [23]. More recently, the *in vitro* and *in vivo* tumor-targeting properties of CTX have been shown to retain following conjugation to a fluorescent dye [24], nanoparticles [25–27], and polymers [28].

We have previously reported CTX-dependent inhibition of proliferation and motility in glioblastoma cells using a targeted bionanocapsule displaying the monomeric fusion protein of chlorotoxin (M-CTX-Fc). Moreover, M-CTX-Fc had a more efficient inhibitory effect on migration than CTX. We observed cellular uptake of the bionanocapsules, indicating M-CTX-Fc is an effective vehicle as a drug delivery system.

MMPs are overexpressed in a variety of malignant tumors, including brain, pancreas, prostate, ovarian, bladder, and lung, and they act as ECM-remodeling enzymes; therefore, targeting of these molecules in cancer therapy is a promising approach to suppress their malignancy. The PANC-1, the human cell line derived from pancreatic carcinoma, is overexpressing MMP-2, MT1-MMP, and MT2-MMP [2]. Thus, the aim of this study was to identify the inhibitory mechanism of M-CTX-Fc on MMP-2 in PANC-1.

## 2. Materials and Methods

### 2.1. Cell Culture

The human cell line derived from pancreatic carcinoma, PANC-1 (RCB2095), and the glioblastoma, A172 (RCB2530), were provided by the National BioResource Project of MEXT, Japan. Human breast carcinoma derived cell line SKBR-3 was obtained from ATCC (Manassas, VA). The cells were grown and subcultured in RPMI medium (Sigma-Aldrich, St. Louis, MO, USA) supplemented with 10% fetal bovine serum (FBS, PAA Laboratories, Pasching, Austria) in the presence of 100 IU/mL penicillin and 100 *μ*g/mL streptomycin (Nacalai Tesque, Kyoto, Japan). The cells were maintained at 37°C in a humidified incubator with 95% air and 5% CO_2_.

### 2.2. Expression and Purification of M-CTX-Fc

The preparation of M-CTX-Fc was performed as previously described [29]. *Escherichia coli* BL21 (DE3) pLysS (Novagen) was transformed with the expression vector for M-CTX-Fc. After induction of the expression vector, the transformant was cultured and the bacteria were harvested. The inclusion bodies were washed and then were dissolved in 6 M guanidinium-HCl containing 0.1 M Tris-HCl (pH 8.5). The protein in the solution was reduced and then refolded. The solution containing refolded protein was purified using a cobalt resin column (Talon Superflow Metal Affinity Resin, Clontech, Mountain View, CA, USA). The eluted solution was dialyzed thrice using phosphate-buffered saline (Dulbecco's formula, hereafter PBS). The purity of M-CTX-Fc in the final preparation was assessed by sodium dodecyl sulphate-polyacrylamide gel electrophoresis (SDS-PAGE), Coomassie Brilliant Blue (CBB) staining, and western blotting.

### 2.3. Preparation of the Conditioned Media for Zymography and Western Blot

PANC-1 cells were seeded at a density of 1.0 × 10^5^ cells per 35 mm dish in RPMI medium supplemented with 10% FBS. After 20 h of culture, the cells were washed with serum-free medium and incubated for an additional 24 h in the same serum-free medium with and without 12, 60, and 300 nM human IgG (Sigma), CTX (AnaSpec Inc., Fremont), and M-CTX-Fc, respectively. The conditioned media (CM) were collected and centrifuged to remove insoluble materials and then stored at −80°C until used in zymography and western blotting.

### 2.4. Gelatin Zymography

MMP-2 gelatinolytic activity was determined in the CM of PANC-1 cells. Fifteen-microliter aliquots of CM were subjected to SDS-PAGE (10%) in the presence of 0.05% gelatin. The samples were not boiled prior to electrophoresis. After electrophoresis, the gels were washed twice in 2.5% Triton X-100 for 30 min and once in 50 mM of Tris-HCl (pH 7.4) for 15 min. The gels were then incubated for 16–20 h at 37°C in buffer A, which contained 30 mM Tris-HCl (pH 7.4), 5 mM CaCl_2_, 0.5 *μ*M ZnCl_2_, 0.2 M NaCl, and 0.02% NaN_3_. After incubation, the gels were stained with Coomassie Brilliant Blue in 50% methanol and 10% acetic acid and destained in 10% methanol and 10% acetic acid.

### 2.5. Quantitative Realtime PCR (qRT-PCR)

PANC-1 cells were untreated or treated with 300 nM M-CTX-Fc for 6 and 24 h. Total RNA was isolated from cells using an RNeasy Minikit (Qiagen). Two micrograms of the total RNA was transcribed with superscript II (Invitrogen) in accordance with the manufacture's protocol. The primer sequences of MMP-2 were forward primer 5′-TTTCCATTCCGCTTCCAGGGCACAT-3′ and reverse primer 5′-TCGCACACCACATCTTTCCGTCACT-3′. qRT-PCR was performed with SYBR Green Realtime Master Mix (Toyobo, Japan) in triplicate containing 5 ng of cDNA along with 400 nM primers using a LightCycler system (TM Roche). The thermal cycling condition was as follows: 95°C for 5 min followed by 45 cycles of 95°C for 10 s, 57°C for 10 s, and 72°C for 12 s.

### 2.6. Biotinylation Assay for the Internalization of M-CTX-Fc

Biotinylation was performed as described previously [30]. PANC-1 cells were plated in a 60 mm tissue culture dish in complete medium. At 90% confluency, the cells were washed twice in Hank's balanced salt solution (HBSS) for 10 min at 4°C. Sulfo-NHS-SS-Biotin (Thermo Scientific), dissolved in HBSS at a concentration of 0.5 mg/mL, was added to the cells at 4°C with mild shaking for 20 min, and this reaction was repeated twice. The cells were washed with HEPES buffered RPMI supplemented with 1% BSA and 2 mM glutamine (RPMI-BSA) for 10 min at 4°C. Control cells were incubated with RPMI-BSA for 1 h at 37°C. The cells were then incubated with either 300 nM M-CTX-Fc or human IgG in RPMI-BSA for 1 h at 4°C or 37°C. The treatment was stopped by placing the dishes back on ice and rinsing the cells twice with HBSS. The biotin on the cell surface was cleaved by incubation of the cells in a reducing solution consisting of 20 mM DTT, 50 mM Tris-HCl (pH 8.7), 100 mM NaCl, and 2.5 mM CaCl_2_ for 20 min at 4°C and was repeated twice. The cells were washed thrice with HBSS, scraped off with lysis buffer consisting of 1% Triton X-100, 50 mM Tris-HCl (pH 7.4), 150 mM NaCl, 5 mM EDTA, 1 mM PMSF, and protease inhibitor cocktail (Sigma-Aldrich), and incubated for 20 min at 4°C. The lysates were collected and sonicated twice, and cell extracts were clarified by centrifugation for 5 min at 4°C. Protein concentrations in the extracts were determined by a BCA assay (Pierce Chemical). Twenty microliters of avidin agarose (Sigma-Aldrich) was added to the extracts, which were incubated overnight at 4°C. After centrifugation for 30 s at 4°C, the agarose was washed thrice in lysis buffer, suspended in Laemmli buffer with *β*-mercaptoethanol, heated for 5 min at 95°C, and finally processed for western blotting. Transferrin receptor internalization was used as a control for immunoprecipitation experiments.

### 2.7. Western Blotting and Image Analysis

One hundred fifty microliters of the CMs was concentrated 10-fold by the methanol/chloroform/water method. The concentrated CM samples were resolved in Laemmli-buffer supplemented with *β*-mercaptoethanol and subjected to SDS-PAGE and western blotting. Proteins resolved on SDS-PAGE were transferred to a polyvinylidene difluoride (PVDF) membrane (Millipore, Billerica, MA, USA). The membrane was blocked with 10% skim milk in 10 mM Tris-HCl (pH 7.4) and 150 mM NaCl containing 0.1% Tween-20 (TBST). To detect MMP-2, the blots were probed with anti-MMP-2 rabbit antibody (Abcam) and anti-rabbit (IgG) goat antibody conjugated with HRP (Cell Signaling Technology, Beverly, MA, USA). To assay biotinylation, the blots were probed with anti-human IgG mouse monoclonal antibody conjugated with HRP (Life Technologies, Carlsbad, CA, USA) diluted to 1 : 1000 in TBST containing 10% skim milk, anti-transferrin receptor mouse monoclonal antibody diluted 1 : 1000 (Invitrogen), followed by anti-mouse IgG horse antibody conjugated with HRP diluted 1 : 2000 (Cell Signaling Technology, Beverly, MA, USA). The HRP signal was developed using a Western Lightning Plus-ECL chemiluminescence reagent (PerkinElmer, Waltham, MA, USA), and the intensities of the bands were visualized using a Light-Capture II cooled CCD camera system (ATTO, Tokyo, Japan). Quantitative assessments of the relative intensity of the blots were analyzed using Image J.

### 2.8. Confocal Microscopic Observation

For confocal microscopic observation, PANC-1 cells were grown on 18 mm cover slips (Iwaki, Tokyo, Japan) in 12-well plates. The cells were incubated with 300 nM M-CTX-Fc [30] in PBS containing 1% BSA for 30 min at 4°C or 37°C. The cells were washed twice with PBS to evaluate specific binding to cell surfaces. The cells were fixed with 4% paraformaldehyde in PBS, permeabilized with 0.2% Triton X-100, and blocked with a blocking solution containing 10% FBS or 1% BSA in PBS. The cells were washed with PBS and incubated with anti-early endosome antigen-1 (EEA-1) antibody (Cell Signaling Technology, Beverly, MA, USA) for 1 h at 25°C followed by Alexa 555-labeled anti-rabbit IgG (Molecular Probes Inc., Eugene, OR, USA) for 30 min at 25°C. The cells were washed with PBS and incubated with FITC-labeled anti-human IgG-Fc antibody (Sigma-Aldrich) for 1 h at 25°C. After further washes, the nuclei were stained with DAPI (Vector Laboratories Inc., Burlingame, CA, USA), and the cells were visualized using a confocal microscope (IX81; Olympus) with Fluoview FV-1000 (Olympus).

### 2.9. Cell Proliferation Assay

The effect on cell proliferation in PANC-1 cells by M-CTX-Fc was evaluated by cell count. PANC-1 cells were seeded onto 12-well plates at a density of 3 × 10^4^ or 1 × 10^5^ cells/well, and cultured in RPMI medium supplemented with 10% FBS. After 20 h of culture, M-CTX-Fc in a range of 0–300 nM were added in triplicate, and the cells were further cultured for 24 h or 48 h. The cells were then trypsinized and counted with TC10 automated cell counter (Bio-Rad, Hercules, CA, USA).

The inhibition of cell growth by human IgG, CTX, and M-CTX-Fc was evaluated using a 3-(4, 5-dimethylthiazol-2-yl)-2, 5-diphenyltetrazolium bromide (MTT) cleavage assay. The cells were seeded at a density of 5 × 10^3^ cells/well in 96-well plates in RPMI medium supplemented with PANC-1 and 10% FBS. After 20 h of culture, human IgG, CTX, and M-CTX-Fc in a range of 0–300 nM was added in triplicate, and the cells were further cultured for 48 h. The cells were then exposed to 5 mg/mL MTT in PBS at a final concentration of 1 mg/mL in culture for 5 h. Formazan crystals formed during the incubation period were dissolved overnight at 37°C by adding 10% SDS containing 20 mM HCl. The absorbance was measured at 570 nm. To assess the viability of cells treated with CTX and M-CTX-Fc after 48 h incubation with different concentrations of CTX and M-CTX-Fc, the wells were washed twice with RPMI medium supplemented with 10% FBS. The cells were further incubated for 48 h in RPMI medium supplemented with 10% FBS. The viable cells were evaluated using the MTT cleavage assay, as described above.

### 2.10. Cell Migration Assay

The migration of PANC-1 cells was assayed in 24-well plates with 8 *μ*m pore cell culture inserts (BD, Franklin Lakes, NJ, USA). Five hundred microliters of RPMI medium supplemented with 10% FBS was added to each well, and 3 × 10^4^ cells were seeded into each insert. The cells were incubated with human IgG, CTX, and M-CTX-Fc, in a range of 0–300 nM in RPMI medium supplemented with 1% BSA at 37°C. After 48 h of culture, the insert chambers were removed and adherent cells on the bottom of each well were counted under microscope. The number of migrated cells was normalized to the number of adherent cells in the absence of human IgG, CTX, and M-CTX-Fc.

### 2.11. Statistical Analysis

The data is expressed as the mean ± SE. The statistical significance of differences between means was determined using Student's *t*-test. Differences were statistically significant at *P* < 0.05.

## 3. Results

### 3.1. PANC-1 Cells Express MMP-2

**Figure 1:**
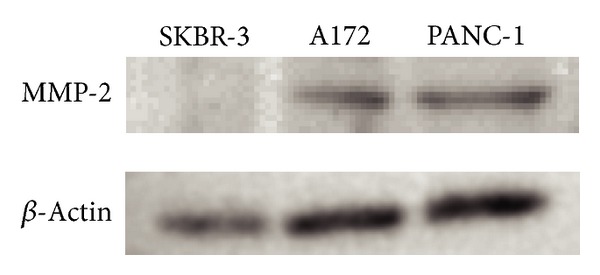
Expression of matrix metalloproteinase-2 (MMP-2) in PANC-1. The protein extracted from SKBR-3 (as a negative control), A172 (as a positive control), and PANC-1 were immunoblotted and detected using anti-MMP2 antibody. *β*-actin was used as loading control.

The 72 kDa protein reacted with anti-MMP2 antibody corresponding to proMMP-2 was observed in PANC-1 and glioma cells (A172) but not in SKBR-3 cells (used as a no/low-expressing control) [31] (Figure 1). A172, in which the expression of MMP-2 is well known, was used as a positive control. Because PANC-1 cells were confirmed to express the MMP-2 protein, we studied the effect of M-CTX-Fc on PANC-1 cells hereafter.

### 3.2. Effect of M-CTX-Fc on the Secretion of MMP-2

**Figure 2:**
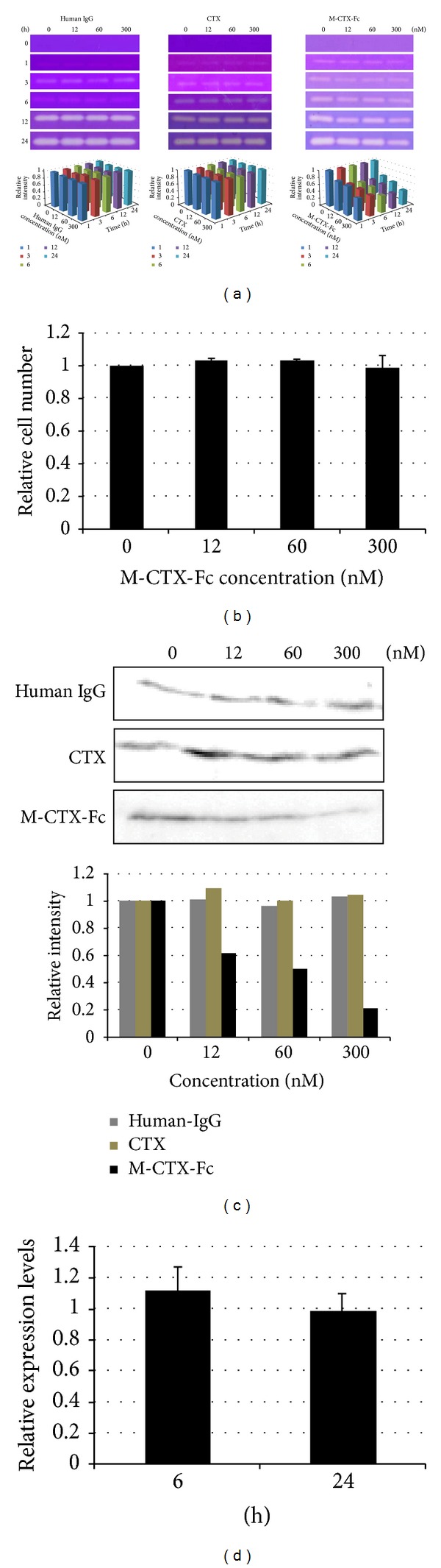
(a) Gelatinase activities of conditioned media (CM) from cultured PANC-1 cells. Cells were treated with 0, 12, 60, and 300 nM human IgG, chlorotoxin (CTX), and M-CTX-Fc at 1, 3, 6, 12, and 24 h. Each 15 *μ*L of the CM were subjected to gelatin zymography. (b) PANC-1 cells were seeded onto 12-well plates at a density of 1 × 10^5^ cells/well and cultured. After 20 h of culture, M-CTX-Fc at a range of 0–300 nM was added to each well for 24 h, and then the cells were trypsinized and counted. (c) M-CTX-Fc decreased MMP-2 release in PANC-1 cells. PANC-1 cells were treated with incremental concentrations of human IgG, CTX, and M-CTX-Fc for 24 h. The CM were collected and concentrated. The protein in the CM was separated using 10% SDS-PAGE, transferred to a PVDF membrane, and probed with monoclonal MMP-2 antibody. (d) qRT-PCR analysis of MMP-2 expression in the absence and presence of M-CTX-Fc. qRT-PCR analysis was performed using primers for MMP-2. Primers specific for GAPDH were used as an internal control. PCR products were separated using 2% TAE agarose gel electrophoresis.

To examine the changes in activity and the expression of MMP-2 in the presence of human IgG (negative control), CTX (positive control), and M-CTX-Fc, gelatinase activity in the CM from PANC-1 cells was analyzed by gelatin zymography. Cells were treated with incremental concentrations of human IgG, CTX, and M-CTX-Fc in a range of 0–300 nM for 1, 3, 6, 12, and 24 h. The effect of human IgG, CTX, and M-CTX-Fc on MMP-2 zymogen in the CM was determined. M-CTX-Fc caused dose-dependent reduction in the amount of zymogen (72 kDa MMP-2 proenzyme) in the CM of PANC-1 cells at 1, 3, 6, 12, and 24 h (Figure 2(a)). In contrast, amount of MMP-2 proenzyme was not significantly affected by human IgG and CTX (Figure 2(a)). The M-CTX-Fc in a range of 0–300 nM did not inhibit the cell proliferation (Figure 2(b)). The result indicated that the inhibition of MMP-2 secretion was not due to M-CTX-Fc cytotoxicity.

The decrease in the amount of MMP-2 was confirmed by western blotting. PANC-1 cells were treated with 12, 60, and 300 nM of human IgG, CTX, and M-CTX-Fc, respectively, for 24 h, and then the CMs were concentrated. The effect of human IgG, CTX, and M-CTX-Fc on MMP-2 protein secretion levels in the CM was determined by western blotting analysis using antibodies recognizing the MMP-2. M-CTX-Fc decreased the levels in the CM of PANC-1 cells in a dose-dependent manner in the range of 0–300 nM. At a concentration of 300 nM M-CTX-Fc, 79% of MMP-2 release was suppressed (Figure 2(c)). In contrast, human IgG and CTX treatment did not cause noticeable variation of MMP-2 levels (Figure 2(c)).

To determine whether the decrease in the MMP-2 protein expression correlates with MMP-2 gene expression, we performed qRT-PCR that showed the MMP-2 gene expression was not significantly affected by M-CTX-Fc in PANC-1 cells (Figure 2(d)). The decrease of the amount of MMP-2 proenzyme secreted into CM may be caused by the decrease in the release of MMP-2 into the CM.

### 3.3. Intracellular Localization of M-CTX-Fc in PANC-1 Cells

**Figure 3:**
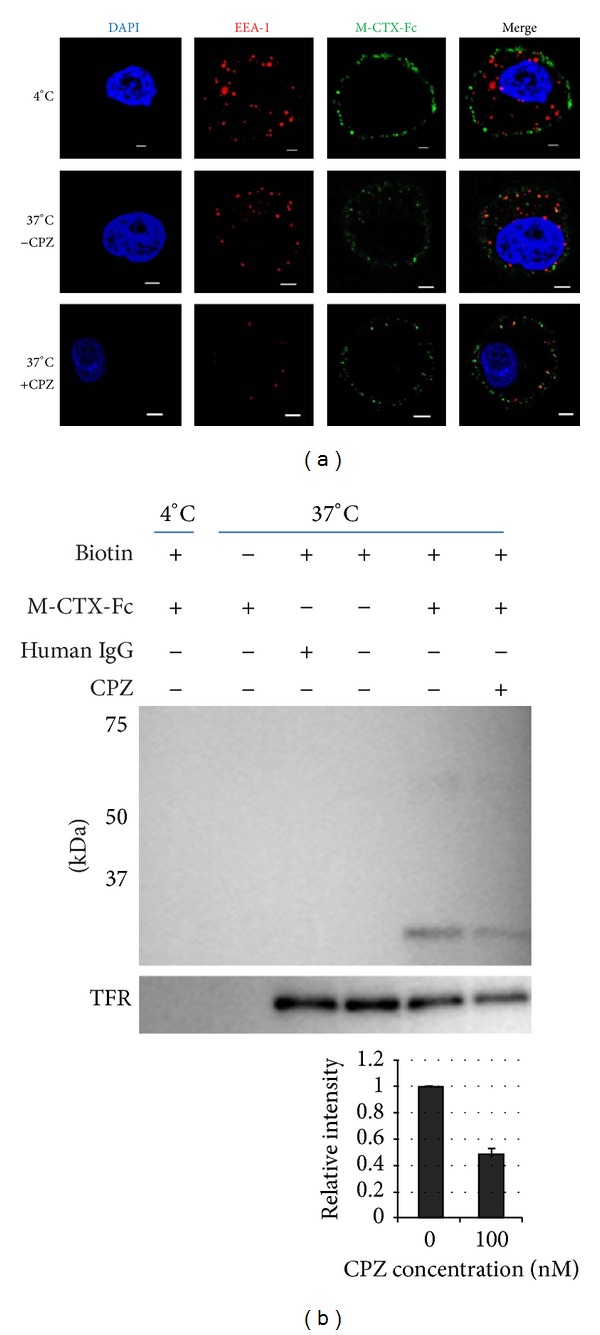
Evaluation of M-CTX-Fc internalized by PANC-1 cells. (a) PANC-1 cells were treated with M-CTX-Fc at 37°C and 4°C and stained with FITC-labeled anti-human IgG antibody and with anti-EEA1 antibody followed by a secondary antibody against Alexa flour 555 labeled-Rabbit IgG. (b) Cell surface receptors were reversibly biotinylated with NHS-SS-Biotin and were incubated with M-CTX-Fc for 1 h. Lane 1: biotinylated cells treated with M-CTX-Fc at 4°C; Lane 2: nonbiotinylated cells treated with M-CTX-Fc at 37°C; Lane 3: biotinylated cells treated with human IgG at 37°C; Lane 4: biotinylated cells left untreated at 37°C for 1 h; Lane 5 and 6: biotinylated cells with M-CTX-Fc at 37°C in the absence and presence of 100 nM CPZ. After treatment, cells were lysed, immunopreciptated with avidin agarose, and subjected to western blot to detect M-CTX-Fc with antihuman IgG antibody. Transferrin was monitored simultaneously as the control for internalization. The intensity of M-CTX-Fc was densitometrically analyzed by ImageJ and plotted to evaluate internalization.

Because of the high expression levels of MMP-2, we evaluated the binding capability of M-CTX-Fc on the surface of PANC-1 pancreatic cells. When the cells were incubated with M-CTX-Fc at 4°C, the fluorescence from FITC-labeled anti-human IgG indicated localization of the fused proteins on the plasma membrane. However, when the cells were incubated at 37°C, the fluorescence indicated that M-CTX-Fc was localized intracellularly in PANC-1 cells (Figure 3(a)).

Because cells adopt divergent pathways for endocytosis, the key pathways are divided into clathrin-dependent and clathrin-independent mechanisms [32]. The clathrin-independent pathways are further classified into caveolar and GPI-anchored early endocytic compartment (GEEC) pathways [32, 33].

The mechanism of M-CTX-Fc uptake was assessed in PANC-1 cells using endocytotic pathway inhibitors. PANC-1 cells were treated with M-CTX-Fc in the presence or absence of inhibitor for clathrin and the internalization was assessed. When the cells were treated with 100 nM CPZ, an amphiphilic drug, which inhibits the clathrin mediated pathway [30], the internalization of the M-CTX-Fc was reduced (Figure 3(a)).

Most of the cargos irrespective of their route merge with Rab5 and early endosome antigen-1 (EEA-1) enriched in early endosomes, which is further sorted into various intracellular destinations [34]. We thus analyzed the recruitment of M-CTX-Fc into early endosomes using an early endosomal marker EEA1 after M-CTX-Fc stimulation in PANC-1 cells at 4°C and 37°C in the presence and absence of CPZ. Localization in endosomes was not observed in cells after 300 nM M-CTX-Fc treatment for 1 h at 4°C. However colocalization of M-CTX-Fc with EEA1 was observed beneath the cell membrane of cells after M-CTX-Fc treatment for 1 h at 37°C which was reduced in the presence of CPZ (Figure 3(a)).

### 3.4. Internalization of M-CTX-Fc

To determine the amount of internalized M-CTX-Fc that was colocalized with EEA1, biotinylation assay was assessed. Surface biotinylated cells were treated with 300 nM M-CTX-Fc and human IgG at 37°C for 1 h. The proteins on cell surface were removed by reduction buffer and washing; then only internalized biotinylated proteins were being assessed. Cell lysates were subjected to precipitation with avidin agarose and blotted against anti-human IgG (Fc-domain specific) antibody conjugated with HRP to analyze the internalized M-CTX-Fc in PANC-1 cells. As for the control, the biotinylated cells were exposed by M-CTX-Fc at 4°C for 1 h. Internalization of the transferrin receptor was also monitored as an endocytosis control.

When the biotinylated cells were treated with 300 nM M-CTX-Fc, the internalization of M-CTX-Fc was increased relative to untreated cells at 37°C (Figure 3(b)). The incubation of cells at 37°C facilitated the intracellular localization of M-CTX-Fc, indicating that the temperature-dependent internalization was attributable to a membrane-dependent mechanism. In contrast, the human IgG produced no internalization at 37°C, indicating specific binding of the CTX moiety to PANC-1 cell surfaces. Without biotinylation, cells incubated with M-CTX-Fc at 37°C produced no signals on western blotting, indicating that the results of immunoprecipitation were detected by biotin-labeling specific reaction (Figure 3(b)).

When the cells were treated with 300 nM of M-CTX-Fc and 100 nM CPZ, the internalization of M-CTX-Fc was reduced. M-CTX-Fc is believed to be internalizing into PANC-1 cells through a clathrin-mediated mechanism.

### 3.5. Effect of M-CTX-Fc on the Migration of PANC-1 Cells

**Figure 4:**
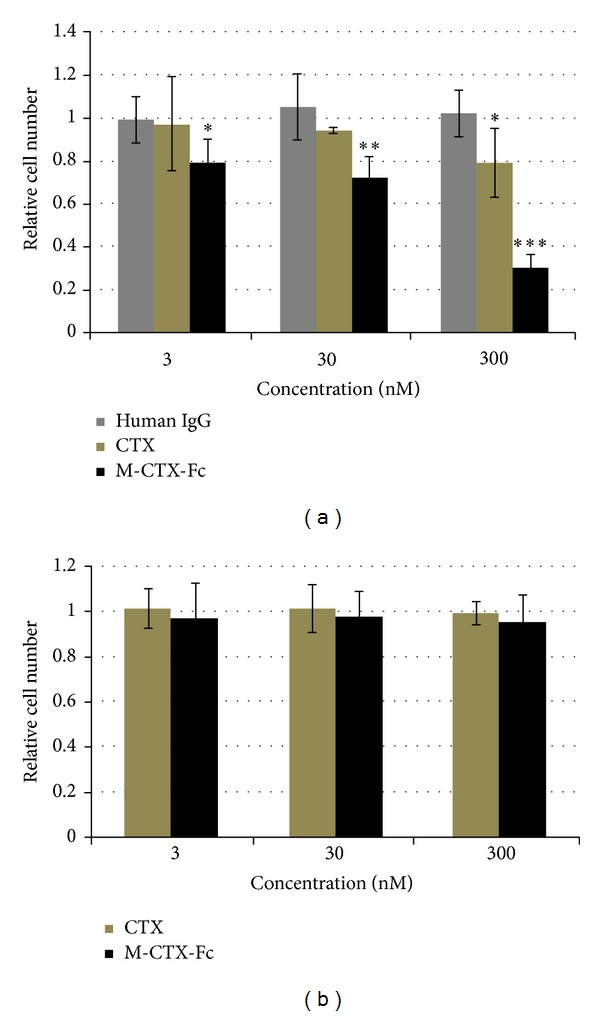
Cell migration assay. (a) The effect of human IgG, CTX, and M-CTX-Fc on the migration of PANC-1 cells was assessed using a PET track-etched membrane culture insert (pore size, 8.0 *μ*m). 3 × 10^4^ cells were incubated with human IgG, CTX, and M-CTX-Fc in the range of 0–300 nM. Translocated cell numbers were normalized against those in the absence of human IgG, CTX, and M-CTX-Fc. The results are shown as mean ± SD from three independent experiments. (*P < 0.1, **P < 0.05, ***P < 0.01). (b) PANC-1 cells were seeded onto 12-well plates at a density of 3 × 10^4^ cells/well and cultured. After 20 h of culture, M-CTX-Fc at a range of 0–300 nM was added to each well for 48 h, and then the cells were trypsinized and counted.

The effect of human IgG, CTX, and M-CTX-Fc on the migration of PANC-1 cells was assessed (Figure 4(a)). Although M-CTX-Fc and CTX at a concentration of 300 nM significantly inhibited the cellular migration, M-CTX-Fc inhibition was clearly concentration dependent. The maximal inhibition obtained with M-CTX-Fc was 70% compared with untreated control cells. The results showed that M-CTX-Fc had a more efficient inhibitory effect than CTX, which was not observed with human IgG at the same concentration. The M-CTX-Fc in a range of 0–300 nM did not inhibit the cell proliferation (Figure 4(b)). The result indicated that the inhibition of cell migration was not due to cell proliferation.

### 3.6. Effect of M-CTX-Fc on the Proliferation of PANC-1 Cells

**Figure 5:**
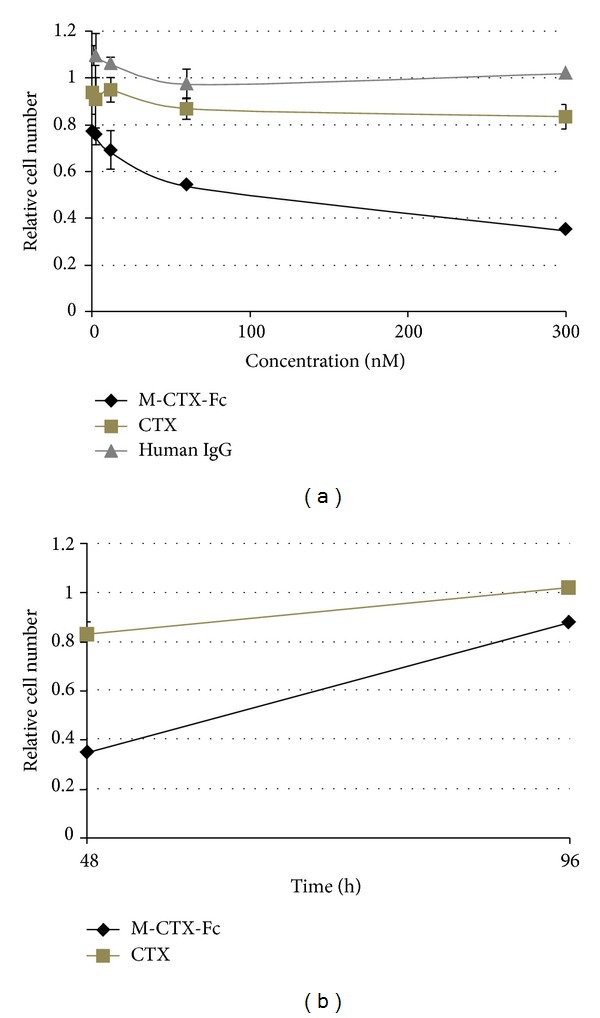
(a) Proliferation inhibition activity. Five thousand cells were grown in the presence of CTX and M-CTX-Fc for 48 h. (b) The viable cells at 48 h were kept cultured without CTX and M-CTX-Fc up to 96 h. Cell numbers in each well were assessed by a 3-(4,5-dimethylthiazol-2-yl)-2,5-diphenyltetrazolium bromide (MTT) assay. The absorbance at 570 nm corresponding to initial number of cells was defined as 1.

We then evaluated the effects of human IgG, CTX, and M-CTX-Fc on the proliferation and viability of PANC-1 cells. M-CTX-Fc strongly suppressed the cell viability compared with CTX (Figure 5(a)). The IC_50_ was estimated at approximately 200 nM. After treatment with 300 nM CTX and M-CTX-Fc for 48 h, the growth of cells was resumed in the next 48 h when the medium was replaced with a medium without CTX and M-CTX-Fc (Figure 5(b)).

## 4. Discussion and Conclusion

CTX is a 36-amino acid peptide that belongs to a large family of insect toxins. Several recent studies have suggested that CTX is a highly specific ligand for malignant human gliomas and shows no significant binding to normal brain tissue [21]. CTX has been shown to bind to a 68–72 kDa membrane protein in glioma cells, where it causes inhibition of transmembrane chloride ion fluxes, presumably by inhibiting Cl^−^ channels. For use as ligands, we designed a fusion protein between CTX and the human IgG-Fc domain, which exists as a 30 kDa monomer.

Matrix metalloproteinases (MMPs), zinc endopeptidases, are capable of proteolysis of numerous ECM components. Over 25 members of this family have been identified to date. MMP-2, -3, -7, -9, -11, and -14 have been evaluated in pancreatic cancer cells [35–37]. The mechanism of activation and regulation of MMP-2 is tightly regulated by several other proteins that form a macromolecular complex specifically involving interactions with membrane-associated MT1-MMP (MMP-14) and **α**_*v*_ **β**_*3*_ integrin, matrix proteins and the endogenous inhibitor of MMP-2, TIMP-2 [38, 39]. MT1-MMP, a membrane type MMP, activates MMP-2, and **α**_*v*_ **β**_*3*_ integrin promotes the maturation and release of MMP-2 [38, 39]. MMP-2 is secreted from the cell in the proform, which is then extracellularly activated to a 62 kDa mature protease at the cell surface by the membrane-bound MT1-MMP and TIMP-2 [40, 41]. In the present study, we demonstrated MMP-2 expression in PANC-1 cells (Figure 1).

We also evaluated the effect of M-CTX-Fc on MMP-2 proteolytic activity by gelatin zymography. The exposure of M-CTX-Fc decreased the amount of MMP-2 zymogen in a dose-dependent manner (Figure 2(a)). We detected the amount of MMP-2 in cultured medium by western blotting analysis. M-CTX-Fc decreased the levels of MMP-2 presented in the CM of PANC-1 cells in a dose-dependent manner (Figure 2(c)). MMP-2, a key mediator of ECM degradation and cell migration, appears to be a target for M-CTX-Fc. Sonthemier et al. identified MMP-2, the membrane type metalloprotease-1 macromolecular complex, and the CLC-3 chloride ion channel as targets for CTX on the surface of human glioma cells [20, 42]. In subsequent studies, Veiseh et al. also demonstrated that MMP-2 facilitates the binding of CTX to MCF-7 breast cancer cells [24]. However, these studies were unable to demonstrate a direct interaction between CTX-Cy5.5 and recombinant MMP-2, suggesting that the molecular target for CTX is yet unknown [24]. Although we could not confirm the binding between M-CTX-Fc and MMP-2, and MT1-MMP, this is consistent with other research [24]. The exact molecular mechanisms for the decrease of MMP-2 levels into the cell media are currently under investigation.

MT1-MMP is able to internalize into the intracellular space, and like other membrane-binding molecules it is regulated by endocytosis because of the functional role of internalization in the cytoplasmic tail [43]. The regulation of the activity and internalization of MT1-MMP are associated with integrin on the surface of endothelial cells [44]. Endocytosis and accumulation of MT1-MMP are mediated by the clathrin-dependent endocytic pathway [43]. M-CTX-Fc was localized to the intracellular space at 37°C (Figure 3(a)) and was reduced by 100 nM CPZ. The internalization of M-CTX-Fc was also shown to be temperature dependent (Figure 3(b)). The human IgG produced no internalization at 37°C, which indicated specific binding of the CTX moiety to the PANC-1 cell surface (Figure 3(b)).

EEA-1 plays a key role in the clathrin-dependent pathway and contains an FYVE finger, which interacts with PI3 K [45, 46]. PI3 K phosphorylates Rab5, which helps EEA-1 to localize to early-endocytic compartments [47]. We observed colocalization of M-CTX-Fc with EEA-1 that corresponds to the preliminary step in the endosomal pathway before transfer to the sorting endosomes (Figure 3(a)).

MMP-2 and MMP-9 expression has been correlated with pancreatic cancer cell invasion [48] and local recurrence rate [49]. As mentioned above, activation of MMP-2 and other MMPs and expression of MT1-MMP and **α**_*v*_ **β**_*3*_ have been shown to correlate with tumor invasion, neovascularization, and metastasis of glioma [50]; melanoma cells both *in vitro* and *in vivo* [51]; and breast cancer [52]. M-CTX-Fc inhibited cell migration in a dose-dependent manner (Figure 4(a)). In summary, M-CTX-Fc was shown to inhibit and arrest the cell proliferation machinery without being toxic to the cells.

The findings presented in this study have significant therapeutic implications. M-CTX-Fc markedly inhibited the migration of PANC-1 suggesting this drug can be useful in the treatment of pancreatic cancer. Furthermore, CTX has passed preclinical safety studies and has recently won FDA approval for use in a phase I/II clinical trial [20]. Several embryologically-related tumors have also been shown to express MMP-2 and to bind CTX [22]. Clinical use of CTX may thus be expanded to include these tumors as well. However, CTX may have even broader utility as a potentially specific MMP-2 inhibitor. MMP-2 is implicated in a range of diseases that involve tissue remodeling in disease progression. Several chemical inhibitors of MMP-2 are in various stages of clinical testing but most have failed because of toxicity or lack of specificity. CTX may be a safer and more specific drug, worthy of further exploration in this context. Moreover, the M-CTX-Fc fusion protein may be an effective instrument for targeting MMP-2-expressing cells and drug delivery.
